# Impact of Engineered Expression of Mitochondrial Association Factor 1b on *Toxoplasma gondii* Infection and the Host Response in a Mouse Model

**DOI:** 10.1128/mSphere.00471-18

**Published:** 2018-10-17

**Authors:** Elizabeth D. English, Jon P. Boyle

**Affiliations:** a Department of Biological Sciences, Kenneth P. Dietrich School of Arts and Sciences, University of Pittsburgh, Pittsburgh, Pennsylvania, USA; Carnegie Mellon University

**Keywords:** mitochondrial association, *Toxoplasma gondii*, chronic infection, cytokines, host-pathogen interactions

## Abstract

The opportunistic intracellular parasite Toxoplasma gondii causes a lifelong chronic infection capable of reactivating in immunocompromised individuals, which can lead to life-threatening complications. Following invasion of the host cell, host mitochondria associate with the parasitophorous vacuole membrane. This phenotype is T. gondii strain specific and is mediated by expression of a host mitochondrial association-competent (HMA^+^) paralog of the parasite protein mitochondrial association factor 1 (MAF1b). Previous work demonstrated that expression of MAF1b in strains that do not normally associate with host mitochondria increases their fitness during acute infection *in vivo*. However, the impact of MAF1b expression during chronic T. gondii infection is unclear. In this study, we assess the impact of MAF1b expression on cyst formation and cytokine production in mice. Despite generally low numbers of cysts generated by the *in vitro* culture-adapted strains used in this study, we find that parasites expressing MAF1b have higher numbers of cysts in the brains of chronically infected mice and that MAF1b^+^ cyst burden significantly increases during the course of chronic infection. Consistent with this, mice infected with MAF1b^+^ parasites have higher levels of the serum cytokines RANTES and VEGF (vascular endothelial growth factor) at day 57 postinfection, although this could be due to higher parasite burden at this time point rather than direct manipulation of these cytokines by MAF1b. Overall these data indicate that MAF1b expression may also be important in determining infection outcome during the chronic phase, either by directly altering the cytokine/signaling environment or by increasing proliferation during the acute and/or chronic phase.

**IMPORTANCE** The parasite Toxoplasma gondii currently infects approximately one-third of the world’s population and causes life-threatening toxoplasmosis in individuals with undeveloped or weakened immune systems. Current treatments are unable to cure T. gondii infection, leaving infected individuals with slow-growing tissue cysts for the remainder of their lives. Previous work has shown that expression of the parasite protein mitochondrial association factor 1 (MAF1b) is responsible for the association of T. gondii parasites with host mitochondria and provides a selective advantage during acute infection. Here we examine the impact of MAF1b expression during chronic T. gondii infection. We find that mice infected with MAF1b-expressing parasites have higher cyst burden and cytokine levels than their wild-type counterparts. A better understanding of the genes involved in establishing and maintaining chronic infection will aid in discovering effective therapeutics for chronically infected individuals.

## Abstract (precis)

The parasite Toxoplasma gondii currently infects approximately one-third of the world’s population and causes life-threatening toxoplasmosis in individuals with undeveloped or weakened immune systems. Current treatments are unable to cure T. gondii infection, leaving infected individuals with slow-growing tissue cysts for the remainder of their lives. Previous work has shown that expression of the parasite protein mitochondrial association factor 1 (MAF1b) is responsible for the association of T. gondii parasites with host mitochondria and provides a selective advantage during acute infection. Here we examine the impact of MAF1b expression during chronic T. gondii infection. We find that mice infected with MAF1b-expressing parasites have higher cyst burden and cytokine levels than their wild-type counterparts. A better understanding of the genes involved in establishing and maintaining chronic infection will aid in discovering effective therapeutics for chronically infected individuals.

## INTRODUCTION

Chronic Toxoplasma gondii infection is marked by the presence of a slow-growing form of the parasite (called the bradyzoite) within cysts that reside primarily in the muscle and central nervous system tissue, including the brain (1, 2). These tissue cysts are refractory to current drug treatments and persist for the life of the infected host (3). Cysts can be found in the brains of infected mice as early as 2 weeks postinfection, and the number of cysts in the brains of infected mice reaches a plateau 4 weeks postinfection (4). The majority of cysts in the brain are spheroidal, and they have a diameter between 5 and 70 μm (2). However, large cysts with a diameter greater than 70 μm are observed, and the number of observed large cysts is greater 5 to 8 weeks postinfection compared to 3 to 4 weeks postinfection (5). Cysts remain intracellular and are surrounded by a glycosylated cyst wall (2, 6).

A number of cyst wall components and other parasite proteins necessary establish or maintain chronic infection have been identified. CST1 is a glycoprotein found within the cyst wall and is responsible for Dolichos biflorus binding to T. gondii cysts (6, 7). Deletion of *CST1* results in a thin and fragile cyst wall, as well as reduced cyst numbers in the brains of infected mice (8). The disruption of the bradyzoite pseudokinase 1 (*BPK1*) gene results in significantly smaller cysts 8 weeks postinfection, and these cysts have a reduced ability to cause oral infection in mice (9). Deletion of genes encoding several T. gondii rhoptry proteins (ROPs) individually or in combination significantly reduces cyst burden in chronically infected mice, specifically ROP2/8, ROP4/7, ROP5, ROP17, ROP18, ROP21, ROP22, ROP25, ROP27, ROP31, ROP35, ROP36, ROP37, ROP38/29/19, ROP40, and ROP41 (10, 11). Together, these studies suggest that the secreted rhoptry kinome plays an important role during chronic infection.

Previous work has established that expression of a host mitochondrial association-competent (HMA^+^) copy of the T. gondii protein, mitochondrial association factor 1 (MAF1b), alters the host cytokine response (12) and provides a competitive advantage during the acute phase of infection in a mouse model (13). At 5 days postinfection (dpi), the serum concentrations of 14 out of 26 cytokines tested (interleukin-10 [IL-10], granulocyte colony-stimulating factor [G-CSF], IL-3, IL-1a, Ccl5, Ccl7, IL-13, IL-4, IL-23, IL-2, IL12p70, interferon gamma [IFN-γ], IL-6, and Ccl3) were reduced in mice infected with a T. gondii RHΔMAF1 mutant compared to mice infected with the wild-type strain (RH:WT) (12), although there were no significant MAF1-dependent differences in mouse morbidity. However, it has also been shown that expression of an HMA^+^ copy of MAF1 (specifically TgMAF1RHb1 [13; GenBank accession no. KU761334], here referred to as MAF1b) in parasites that do not normally exhibit HMA provided a competitive advantage in a mixed population of HMA^+^ and HMA^−^ parasites during the acute phase of infection (13), suggesting that in the type II genetic, background, MAF1b expression (and therefore HMA itself) can influence infection outcome *in vivo*.

Here we aimed to assess the impact of MAF1b complementation in naturally HMA^−^ parasites on the progression and maintenance of chronic infections in mice and the cytokine responses that occur during the progression of disease over a 4-week period of infection. Recently published *in vivo* dual-transcriptional profiling of T. gondii and host (in this case, mouse) RNA, which is available at ToxoDB.org, suggests that *MAF1* expression remains high (>80th percentile) during chronic infection (14). This is consistent with additional data sets both *in vivo* (15) and *in vitro* (ToxoDB.org). It is therefore possible that MAF1b expression and/or MAF1b-mediated HMA impacts parasite biology during chronic infection. Here we provide evidence that mice infected with MAF1b-expressing parasites have higher cyst burdens and altered serum cytokine levels compared to those infected with its wild-type counterpart.

## RESULTS

### Exogenous MAF1b expression in type II parasites increases cyst burden of chronically infected CBA/J mice 4 and 8 weeks postinfection.

**FIG 1:**
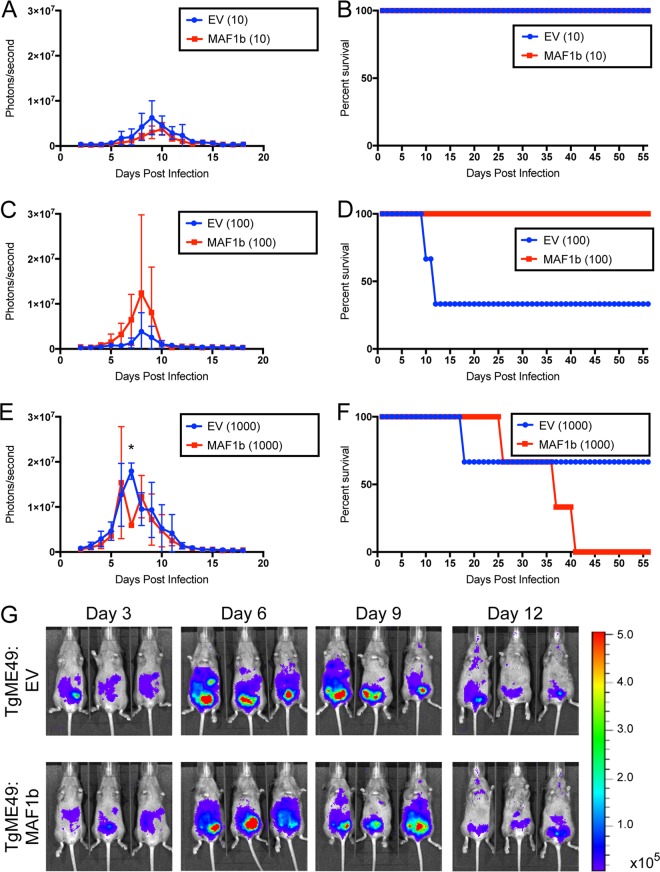
MAF1b expression does not significantly alter parasite burden or mouse survival during acute infection in CBA/J mice. Female CBA/J mice were infected with 10, 100, or 1,000 parasites of TgME49:EV or TgME49:MAF1b, and acute infection was monitored by bioluminescence imaging. (A, C, and E) Quantification of bioluminescence in mice infected with 10, 100, or 1,000 parasites. There was no significant difference in bioluminescence between TgME49:EV (EV)- and TgME49:MAF1b (TgMAF1RHb1)-infected mice at any time at any dose, with the exception of the time point 7 dpi for mice infected with 1,000 parasites, where mice infected with TgME49:EV had significantly higher bioluminescence than mice infected with TgME49:MAF1b (*, *P* = 0.0004). (B, D, and F) Survival curves of mice from panels A, C, and E. There were no significant differences in survival at any dose. (G) Images of infected mice quantified in E on 3, 6, 9, and 12 dpi.

Previous work used a BALB/c mouse model to determine that MAF1b expression in type II T. gondii and the resulting HMA provide a growth advantage during acute infection (13). It is difficult to assess chronic infection in a BALB/c mouse, as chronic infection in BALB/c mice results in low numbers of brain cysts (16). We therefore used a CBA/J mouse model, as CBA/J mice have higher numbers of brain cysts during chronic infection (17, 18). We first performed a dose curve with type II (TgME49:EV [empty vector]) and MAF1b complemented type II parasites (TgME49:MAF1b) in CBA/J mice to determine an appropriate sublethal dose. CBA/J mice were infected with 10, 100, or 1,000 parasites of either TgME49:EV or TgME49:MAF1b, and acute infection was assessed (3 mice per strain per dose). Similar numbers of viable parasites for each strain were confirmed by plaque assay (TgME49:EV, 18/100; TgME49:MAF1b, 23/100), and parasite burden was monitored daily using bioluminescence. As previously observed in BALB/c mice (13), there was no significant difference in parasite burdens between TgME49:EV- and TgME49:MAF1b-infected mice during acute infection at any dose (Fig. 1), with the exception of a single time point, 7 dpi at a dose of 1,000 parasites, for which mice infected with TgME49:EV had significantly higher bioluminescence than mice infected with TgME49:MAF1b (*P* = 0.0004). Although mice became moribund at both the 100- and 1,000-parasite doses, there was no significant difference in survival rates between TgME49:EV- and TGME49:MAF1b-infected mice (Fig. 1).

To assess the impact of MAF1 expression on chronic infection, CBA/J mice were infected with 1,000 parasites of either TgME49:EV or TgME49:MAF1b, and infection was allowed to progress to the chronic stage (10 mice per strain). Mice were then euthanized at the indicated times, and brains were removed in order to quantify cyst burden. Cyst burden was quantified for half the mice at 28 dpi and at 56 (experiment 1) or 60 (experiment 2) dpi for the remaining half. Cyst numbers were generally low compared to those of published studies (5, 10), which may be a result of a high number of passages in cell culture for this particular parental strain. However, cyst numbers were consistent within treatment groups, and each experiment was conducted with independently generated TgME49:MAF1b and TgME49:EV clones to control for variation due to MAF1b insertion location and passage history.

**FIG 2:**
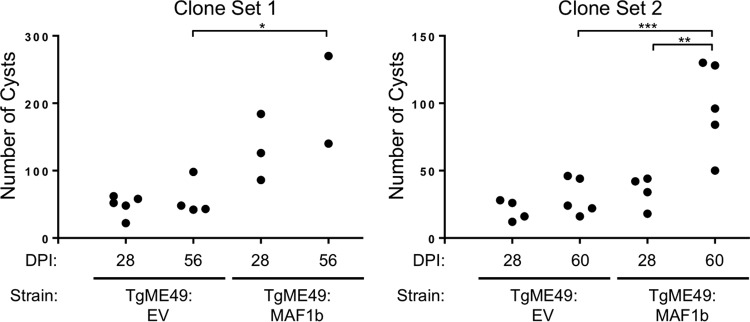
MAF1b expression increases cyst burden in chronically infected CBA/J mice. Female CBA/J mice were infected with 1,000 parasites of TgME49:EV or TgME49:MAF1b. Parasite viability was confirmed by plaque assay (experiment 1, TgME49:EV, 382/1,000, and TgME49:MAF1b, 404/1,000; experiment 2, TgME49:EV, 271/1,000, and TgME49:MAF1b, 364/1,000). Half of each group was sacrificed at 28 dpi, and the remainder were sacrificed 56 (experiment 1) or 60 (experiment 2) dpi. Brains were removed from sacrificed mice and divided sagitally. The left half of each brain was homogenized, and cysts were isolated by Percoll gradient before being stained with rhodamine-conjugated Dolichos biflorus agglutinin. Cysts were then quantified using an inverted fluorescence microscope. Mice infected with TgME49:MAF1b had a higher cyst burden than mice infected with TgME49:EV. This difference was significant 56 or 60 dpi (*, *P* = 0.007; ***, *P* = 0.0005). There were also significantly more cysts 60 dpi compared to 28 dpi in TgME49:MAF1b-infected mice in experiment 2 (**, *P* = 0.0012).

For both experiments, there was a significantly higher cyst burden in mice infected with TgME49:MAF1b compared to mice infected with TgME49:EV (Fig. 2). For the first experiment, mice infected with TgME49:EV had an average of 48.4 cysts (standard deviation [SD], 15.7; *n* = 5) at 28 dpi and 57.8 cysts (SD, 27; *n* = 4) at 56 dpi, while mice infected with TgME49:MAF1b had an average of 132 cysts (SD, 49.3; *n* = 3) at 28 dpi and 205 cysts (SD, 91.9; *n* = 2) at 56 dpi. An ordinary one-way analysis of variance (ANOVA) followed by multiple comparisons (comparing only within a time point or parasite treatment for a total of 4 comparisons) with a Holm-Sidak correction found that in this experiment, the difference in cyst burdens was significant between TgME49:EV- and TgME49:MAF1b-infected mice at 56 dpi (*P* = 0.007). For the second experiment, mice infected with TgME49:EV had an average of 20.5 cysts (SD, 7.7; *n* = 4) at 28 dpi and 30.4 cysts (SD, 13.7; *n* = 5) at 60 dpi, while mice infected with TgME49:MAF1b had an average of 34.5 cysts (SD, 11.8; *n* = 4) at 28 dpi and 97.6 cysts (SD, 33.3, *n* = 5) at 60 dpi. Following an ordinary one-way ANOVA and multiple comparisons, we found that once again, the difference in cyst burdens between TgME49:EV- and TgME49:MAF1b-infected mice was significant at 60 dpi (*P* = 0.0005). Additionally, for this experiment the difference in cyst burdens of TgME49:MAF1b-infected mice was significant between 28 and 60 dpi (*P* = 0.0012).

### Serum cytokine levels differ between mice infected with wild-type type II parasites and type II parasites expressing MAF1b throughout infection.

Previous work has shown that the host cytokine response is elevated during the acute stage of T. gondii infection when MAF1b is expressed (12). To determine if the host response is altered throughout infection, we infected C57BL/6J mice with 1,000 parasites of TgME49:EV or TgME49:MAF1b and collected blood samples during acute and chronic infection (10 mice per strain). For this experiment, C57BL/6J mice were used as they are susceptible to chronic T. gondii infection (17, 18) and C57BL/6J is the background strain for the vast majority of knockout mouse strains. Serum cytokine levels were measured by Luminex 0, 7, 21, 28, and 57 dpi. Two mice appeared to be uninfected, as there was no observed bioluminescence during imaging and there was no interferon gamma (IFN-γ) response, so these mice were eliminated from statistical analyses and graphical representation (one mouse injected with TgME49:EV in the group that was euthanized at 57 dpi and one mouse injected with TgME49:MAF1b in the group that was euthanized at 28 dpi).

We conducted a two-way ANOVA of all 32 cytokines and then applied a Bonferroni’s correction to the ANOVA *P* values (see Table S1 in the supplemental material). Of the 32 cytokines, six had *P* values of <0.05 for the parasite strain type factor after the Bonferroni correction. For these six cytokines (G-CSF, IL-6, MIP-1b, RANTES, tumor necrosis factor alpha [TNF-α], and vascular endothelial growth factor [VEGF]), we conducted multiple comparisons with a Holm-Sidak correction to compare cytokine levels of TgME49:EV-infected mice to TgME49:MAF1b-infected mice for each time point. G-CSF, IL-6, MIP-1b, and TNF-α were each significantly different at 7 dpi, with higher expression in TgME49:MAF1b-infected mice (G-CSF, *P* < 0.0001; IL-6, *P* < 0.0001; MIP-1b, *P* = 0.0007; TNF-α, *P* < 0.0001). For RANTES, TgME49:MAF1b-infected mice had significantly higher expression 28 (*P* = 0.0072) and 57 (*P* = 0.0070) dpi. There was also significantly higher VEGF expression in TgME49:MAF1b-infected mice at 57 dpi (*P* < 0.0001).

10.1128/mSphere.00471-18.1 TABLE S1 Summary of ANOVA applied to cytokines measured by Luminex. Download Table S1, PDF file, 0.1 MB. Copyright © 2018 English and Boyle. 2018 English and Boyle This content is distributed under the terms of the Creative Commons Attribution 4.0 International license.

**FIG 3:**
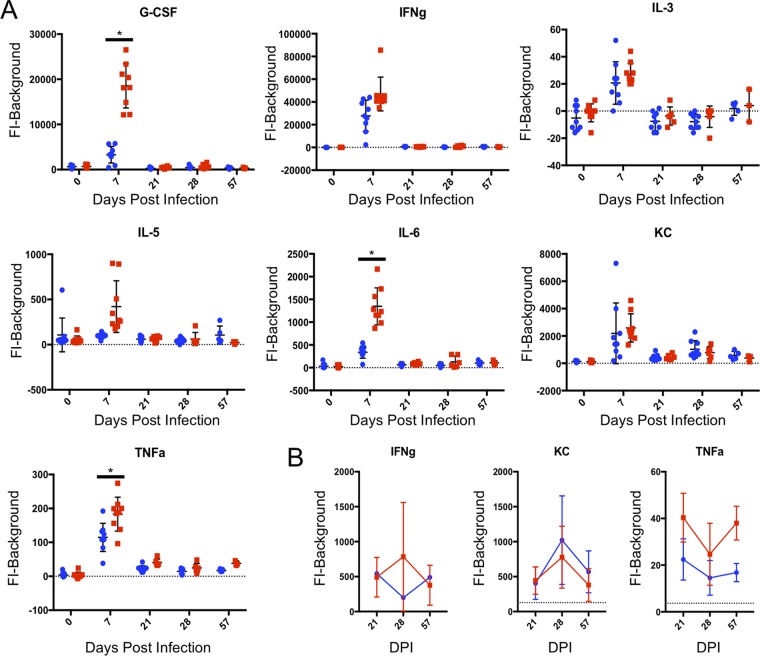
Seven cytokines are elevated during acute infection and return to near preinfection levels during chronic infection. Female C57BL/6J mice were infected with 1,000 parasites of TgME49:EV or TgME49:MAF1b. Blood samples were taken prior to infection, as well as 7, 21, 28, and 57 days postinfection and processed to obtain serum samples. Serum samples were then analyzed by Luminex for the presence of 32 mouse cytokines. For each cytokine, serum levels are given as fluorescent intensity (FI) minus background. Each point represents a single mouse: blue circles indicate mice infected with TgME49:EV, and red squares indicate mice infected with TgMe49:MAF1b. (A) FI minus background for 0, 7, 21, 28, and 57 dpi of seven cytokines elevated during acute infection, but returning to near prebleed levels during chronic infection. Time points where expression was significantly different between TgME49:MAF1b- and TgME49:EV-infected mice are indicated by asterisks. (B) A closer look at 21, 28, and 57 dpi for three of the seven cytokines that were still expressed at low levels during chronic infection. The gray dotted line indicates the average for 0 dpi.

**FIG 4:**
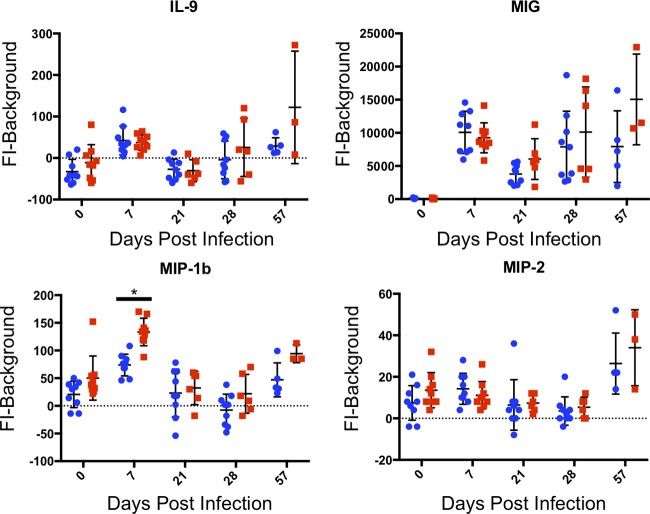
Cytokines expressed during acute infection are expressed at a low level during early chronic infection and then increase in expression throughout the remainder of chronic infection. For each cytokine, serum levels are given as FI minus background. Each point represents a single mouse: blue circles indicate mice infected with TgME49:EV, and red squares indicated mice infected with TgME49:MAF1b. A time point where expression was significantly different between TgME49:EV- and TgME49:MAF1b-infected mice is indicated by an asterisk.

We then examined the cytokine data for general expression patterns regardless of whether or not they had significant differences between parasite strains. For 15 of the 32 cytokines, all Luminex measurements remained at levels similar to those in the prebleed serum (see Table S2 in the supplemental material), and we therefore categorized them as “undetectable.” Seven cytokines (G-CSF, IFN-γ, IL-3, IL-5, IL-6, KC, and TNF-α) were elevated at 7 dpi, but then returned to near prebleed levels of expression by 21 dpi (Fig. 3A). All seven were higher in TgME49:MAF1b-infected mice compared to TgME49:EV-infected mice; however, as stated above only G-CSF, IL-6, and TNF-α expression were significantly different at 7 dpi (*P* < 0.0001). It should be noted that for IFN-γ, KC, and TNF-α, we are able to measure these cytokines in the serum at levels greater than prebleed at 21, 28, and 57 dpi; however, the level of expression is low compared to expression during acute infection (Fig. 3B). Four additional cytokines (IL-9, MIG, MIP-1b, and MIP-2) are elevated at 7 dpi and decrease in expression 21 dpi, but then increase in expression from 21 to 57 dpi (Fig. 4). As previously stated, MIP-1b is expressed at significantly higher levels in TgME49:MAF1b-infected mice compared to TgME49:EV-infected mice at 7 dpi (*P* = 0.0007), but no other time point was statistically significant.

10.1128/mSphere.00471-18.2 TABLE S2 Cytokines measured at or near prebleed levels during mouse infection with TgME49:EV or TgME49:MAF1b. Download Table S2, PDF file, 0.1 MB. Copyright © 2018 English and Boyle. 2018 English and Boyle This content is distributed under the terms of the Creative Commons Attribution 4.0 International license.

**FIG 5:**
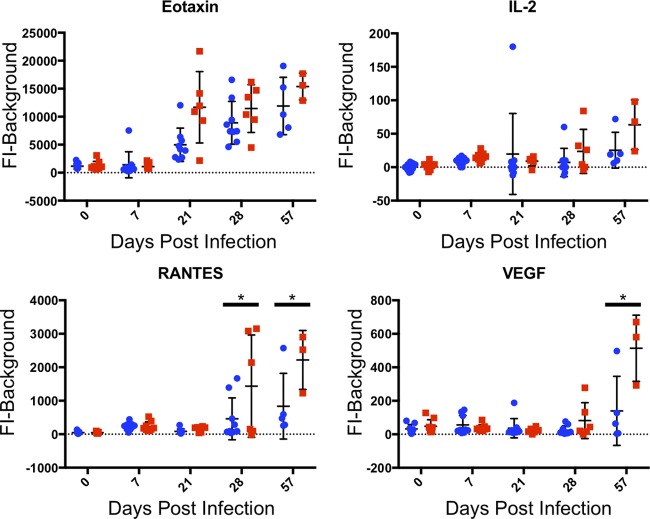
Cytokines expressed during chronic infection. For each cytokine, serum levels are given as FI minus background. Each point represents a single mouse: blue circles indicate mice infected with TgME49:EV, and red squares indicated mice infected with TgME49:MAF1b. Time points where expression was significantly different between TgME49:EV- and TgME49:MAF1b-infected mice are indicated by asterisks.

**FIG 6:**
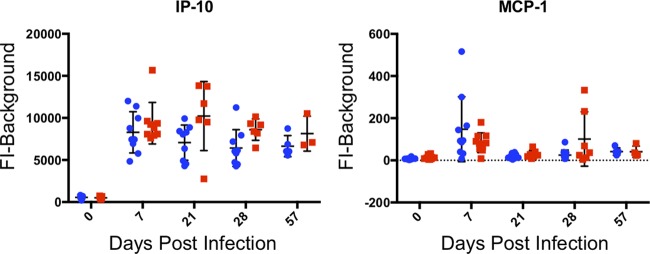
Cytokines with unique expression patterns. For each cytokine, serum levels are given as FI minus background. Each point represents a single mouse: blue circles indicate mice infected with TgME49:EV, and red squares indicated mice infected with TgME49:MAF1b.

Four more cytokines (eotaxin, IL-2, RANTES, and VEGF) remained relatively low during acute infection, and then expression increased throughout chronic infection (Fig. 5). Generally, TgME49:MAF1b-infected mice had higher levels of all four of these cytokines compared to TgME49:EV-infected mice, and this difference was significant for RANTES at 28 and 57 dpi, as well as for VEGF at 57 dpi. The remaining two cytokines (IP-10 and MCP-1) did not fall into any of the previous categories (Fig. 6). For IP-10, expression was measured above prebleed levels during acute infection and remained at similarly high levels throughout chronic infection. MCP-1 was measured above prebleed levels at 7 dpi, expression returned to near prebleed at 21 dpi, but then was measured above prebleed levels again at 28 dpi, and returned to prebleed by 57 dpi. At 7 dpi, MCP-1 was higher in TgME49:EV-infected mice, but then at 28 dpi, expression was higher in TgME49:MAF1b-infected mice. These differences were not significant.

## DISCUSSION

Here we provide evidence that MAF1b expression may play a role in the establishment and maintenance of chronic T. gondii infection. Type II parasites, such as the TgME49 strain, do not normally express a copy of MAF1 capable of mediating HMA (specifically the “B” paralogs [13]). TgME49 parasites are quite capable of establishing chronic infection and forming tissue cysts in the brains of infected mice. However, the exogenous expression of an HMA-competent copy of MAF1 (TgMAF1RHb1) leads to a higher number of cysts in the brains of chronically infected mice compared to mice infected with wild-type type II parasites. The observed difference in cyst burden could be due to the slight increase in HMA^+^ parasite growth during acute infection, leading to the establishment of more cysts. However, there is also a significant increase in cyst burden from 4 to 8 weeks postinfection only in mice infected with TgME49:MAF1b (but not TgME49:EV). This suggests that, in addition to its role during the acute phase of infection, MAF1b also plays a role in cyst formation, growth, and/or stability, at least during days 28 to 56 postinfection.

It has traditionally been thought that bradyzoites within tissue cysts are limited in their replicative capabilities. However, a recent study looking directly at parasite division within tissue cysts *in vivo* has shown that bradyzoites replicate both asynchronously and synchronously within mature cysts (5). Our data showing an increase in cyst burden of mice infected with TgME49:MAF1b over time also support the idea that bradyzoites are capable of growth, possibly via replication of parasites within cysts. However, the mechanism by which the cyst burden of TgME49:MAF1b-infected mice increases between days 28 and 56 remains unknown. Further investigations into the mechanism of increased cyst burden over time in these mice will be limited by the lack of technology available to track chronic infection *in vivo*. Staining for T. gondii inner membrane complex protein 3 (TgIMC3) and examination of replication of bradyzoites within cysts may be useful in determining if MAF1b-expressing parasites have altered replication patterns *in vivo* compared to wild-type type II bradyzoites (5). Inducible deletion (via inducible *cre*-mediated recombination systems, for example [19, 20]) of the entire MAF1 locus in mice would also help to determine how MAF1b expression mediates changes in cyst size and/or bradyzoite replication.

In addition to a higher number of brain cysts, mice chronically infected with TgME49:MAF1b have generally elevated cytokine levels compared to the TgME49:EV-infected controls. Cytokines fall broadly into six categories based on their temporal and strain-specific expression profiles. For nearly half of the cytokines (15 of 32 [Table S1]), detection in the serum remained at levels similar to those in the samples collected prior to T. gondii infection. Of the remaining 17 queried cytokines, 13 have expression higher than prebleed levels during acute infection (Fig. 3A, 4, and 6), including several that have been previously identified as being induced during T. gondii infection (G-CSF, IL-3, IL-6, and IFN-γ [12]). Remarkably, for the 13 cytokines that were expressed during acute T. gondii infection, 12 were higher in mice infected with TgME49:MAF1b compared to TgME49:EV. Seven of the 13 cytokines expressed during acute infection return to preinfection levels by 28 dpi, suggesting a primary role in responding to and/or controlling acute infection. It is important to note that of these seven, three are still expressed at low levels during chronic infection (IFN-γ, KC, TNF-α [Fig. 3B]), and for IFN-γ, it has been shown that this cytokine is key for control of chronic T. gondii infection (21). An additional six cytokines expressed during acute infection are also expressed during chronic infection, and in fact for MIG, MIP-2, and IL-9, peak expression is at 57 dpi (Fig. 4). It is possible that these cytokines play roles during both acute and chronic T. gondii infection. MIG, MIP-1b, and MIP-2 are chemoattractants, responsible for recruiting T lymphocytes and granulocytes to sites of inflammation (22–24). These cytokines may be expressed in order to attract host immune cells to the tissues with parasites in order to promote the recognition and clearance of T. gondii. IL-9 is a T-lymphocyte growth factor that is also involved in antiapoptotic signaling via the JAK/STAT pathway (25, 26). Like MIG, MIP-1b, and MIP-2, it is possible that IL-9 plays a role in attracting the appropriate immune response to the site of T. gondii infection.

Interestingly, MCP-1 expression is higher than prebleed at both 7 and 28 dpi. It is higher in TgME49:MAF1b-infected mice at 28 dpi, contrary to its expression profile at 7 dpi, which is higher in TgME49:EV-infected mice (Fig. 6). MCP-1 recruits monocytes, T lymphocytes, and dendritic cells to areas of tissue damage or inflammation (27, 28). Given the timing of expression, it is possible that MCP-1 is expressed in response to tissue damage during acute infection (7 dpi) and chronic infection (28 dpi). It is also possible that the higher expression of MCP-1 observed in TgME49:MAF1b-infected mice at 28 dpi is related to higher levels of tissue damage due to the presence of more parasites in the tissues of these mice during chronic infection.

The final four cytokines examined by Luminex were expressed only during chronic infection, and this expression increased from 21 to 57 dpi (eotaxin, IL-2, RANTES, VEGF [Fig. 5]). Expression of these cytokines was generally higher in TgME49:MAF1b-infected mice compared to TgME49:EV-infected mice, and this difference was significant for RANTES at 28 and 57 dpi and for VEGF at 57 dpi. Eotaxin is involved in aging (29), and it is possible that the increase in eotaxin throughout chronic infection is due to the fact that the mice are aging throughout the experiment. It is possible that IL-2, RANTES, and VEGF are involved in responding to chronic infection.

Overall, when MAF1b is expressed in TgME49 parasites, infected mice express several proinflammatory cytokines (IFN-γ, IL-6, RANTES, TNF-α, and VEGF) at higher levels than mice infected with wild-type TgME49 parasites. A similar increase in expression is not seen with any of the anti-inflammatory cytokines examined by Luminex (IL-4, IL-10, and IL-13). These data are consistent with previous work showing a decrease in inflammatory cytokine production in RHΔ*MAF1* parasite-infected mice compared to those infected with wild-type RH (12), even though *MAF1* deletion had little impact on acute mortality (12). Together, these data suggest that MAF1b expression (and HMA) increase inflammatory responses, particularly during chronic infection. It is possible that these are due to parasite burden differences between these strains (13) and/or host immune responses specifically induced by MAF1b. Given the clear link between mitochondrial biology and innate immunity (30) and the tight association that is observed between MAF1b-expressing T. gondii and the host mitochondria (12, 13, 31), it is not unreasonable to propose that some of the inflammatory responses to HMA^+^ T. gondii are due to MAF1b-mitochondrion interactions. More work with TgMAF1b and TgMAF1a paralogs (and chimeras between them) will help to further genetically dissect the contributions of MAF1 expression and HMA to observed host responses.

We have shown previously that there is a selective advantage to MAF1b expression (13); however, type II strains do not express MAF1b, raising the question as to how the MAF1b-null allele of the *MAF1* locus became fixed in the type II lineage. One possible explanation can found in what is known about other loci unique to T. gondii type II strains and how they may impact host inflammatory responses. There is likely to be an evolutionary trade-off between the growth-enhancing effects of MAF1b expression and the proinflammatory response that it induces. While induction of higher levels of inflammation may serve to reduce parasite growth and dissemination, it can also lead to death of the host (32), an endpoint in T. gondii infection that is likely maladaptive given the fact that T. gondii tissue cysts are not environmentally stable (33, 34). Type II parasites (which are naturally HMA^−^) activate the NF-κB pathway via the secreted protein TgGRA15 (35), which leads to the activation of inflammatory and antiapoptotic pathways (35). Type II parasites also have an allele of the secreted kinase TgROP16, which is less effective than the type I and III alleles at downregulating IL-12 expression via STAT3/6 activation (36). It is possible that the resulting inflammatory response due to the combination of NF-κB activation and reduced STAT3/6 activation provided the selective pressure necessary to select for parasites with reduced (or null) MAF1b expression (while maintaining MAF1a expression [13]) in a *MAF1* locus ancestral to present day type II strains. These questions can be addressed using existing genetic tools in T. gondii and highlight the fact that sexual pathogens like T. gondii harbor multiple loci that contribute to virulence and pathogenicity in different ways. Selection likely operates on all of these loci simultaneously to appropriately balance parasite burden, while preserving the health of the host to allow effective transmission. *MAF1b* may be another locus in the T. gondii arsenal that is capable of mediating this critical balance.

## MATERIALS AND METHODS

### Parasite strains and maintenance.

Parasites were maintained by serial passage in human foreskin fibroblasts (HFFs) at 37°C and 5% CO_2_. HFFs were grown in Dulbecco’s modified Eagle’s medium (DMEM) supplemented with 10% fetal bovine serum, 2 mM glutamine, and 50 μg/ml each of penicillin and streptomycin (CDMEM).

### Generation of constructs and transgenic parasites.

Generation of pMAF1RHb1 (N-terminally hemagglutinin [HA]-tagged MAF1RHb1) expression construct has been described previously (12). Transgenic parasite lines were generated by transfecting the TGME49Δ*hpt*:Luc (MΔLuc) parental strain with 50 µg of HindIII-linearized pMAF1RHb1 or the empty vector pGra-HA_HPT. Stable expression lines were isolated by selection in mycophenolic acid (MPA)/xanthine followed by limiting dilution in 96-well plates. Transgenic parasites were maintained as passage-matched clones.

### Animal experiments.

Mouse experiments were performed with 4- to 8-wk-old female BALB/c, CBA/J, or C57BL/6J mice obtained from Jackson Laboratories. All animal procedures in this study meet the standards of the American Veterinary Association and were approved locally under IACUC protocols 12010130 and 15015428.

### Mouse infection and bioluminescence imaging.

Unless otherwise stated, all infections were achieved by intraperitoneal (i.p.) injection of quantified tachyzoites suspended in 200 μl of phosphate-buffered saline (PBS). Briefly, well-infected flasks were washed with fresh CDMEM, and then monolayers were scraped from the bottom of the flask and syringe lysed by serial passage through a 27- and then 25-gauge needle. Lysed parasites were pelleted by centrifugation at 800 × *g* for 10 min, CDMEM was aspirated, and the pellet was resuspended in 1 to 3 ml PBS. Parasites were then quantified by counting on a hemocytometer and diluted with PBS to the appropriate number of parasites per 200 μl.

Bioluminescence imaging was conducted as previously described (37). Briefly, mice were i.p. injected with 200 μl of 15.4 mg/ml d-luciferin in PBS prior to anesthetization. Ten minutes after injection, mice were imaged dorsally or ventrally using an IVIS Lumina II. Images were analyzed using Living Image software.

### Serum collection.

Blood samples were collected by tail or submandibular bleeding at specified time points. For each sample, 20 to 50 μl of whole blood was collected, incubated on ice for 1 h, and centrifuged at 2,500 *× g* for 10 min. Alternatively, blood was collected into serum separator tubes, allowed to clot for 5 to 10 min, and then centrifuged at 10,000 × *g* for 10 min. In all cases, serum supernatant was transferred to a new tube for further analysis, and the pelleted clot was discarded. For final time points, blood was collected after euthanasia by cardiac puncture for a total volume of 200 to 500 μl and incubated for 1 h on ice, and serum was collected as described above.

### Analysis of cytokine expression.

Serum was collected as described above and stored at −80°C until sent for Luminex bead array analysis. Samples were analyzed by the UPCI Cancer Biomarkers Facility: Luminex Core Laboratory (supported in part by award P30CA047904) for the presence of 32 mouse cytokines. The serum level of each cytokine was reported as fluorescence intensity minus background, and cytokine levels were analyzed using GraphPad PRISM software. Mice were grouped by parasite strain, and each mouse had between one and four time points used in the analysis. A two-way ANOVA was performed using Graph Pad Prism 7 statistical software, with the experimental design set to no matching, as missing data points from mice euthanized during the experiment would not allow for the use of a repeated measures analysis. A Bonferroni correction of 32 was applied to the *P* values obtained. For cytokines with significant *P* values for the column factor (parasite strain) the ANOVA was followed by multiple comparisons with a Holm-Sidak correction between the means of mice infected with TgME49:EV or TgME49:MAF1b at each time point to determine significant differences between serum cytokine levels.

### Cyst purification, staining, and quantification.

Mice were euthanized, and whole brains were removed. Either the entire brain or the left half was stored in PBS with 3% fetal bovine serum (FBS) on ice and then at 4°C until processing. Brain tissue was homogenized by passage through a 100-μm-pore nylon cell strainer and suspended in 3% FBS in a 50-ml conical tube. Brain homogenate was pelleted by centrifugation at 1,500 *× g* for 15 min and 4°C. Excess 3% FBS was removed, leaving ∼5 ml of 3% FBS and brain homogenate. Cysts were then purified using a Percoll gradient of 5 ml each of 90%/40%/20% Percoll diluted in PBS layered into a new 50-ml conical tube (5). Brains were resuspended in PBS/FBS to a total volume of 5 ml, passaged twice through an 18-gauge needle, and then layered dropwise on top of the gradient. Gradients were then subjected to centrifugation at 1,500 *× g* for 15 min at 4°C. Following centrifugation, the top layer of brain tissue and the 90% Percoll layers were removed. An additional 3% FBS was added to the remaining Percoll and 3% FBS layers, disrupting the gradient and bringing the total volume to 45 ml. The tubes were inverted 2 to 3 times to ensure complete disruption of the gradient before centrifugation at 1,500 *× g* for 15 min at 4°C. The top 40 ml was then removed, leaving 5 ml of 3% FBS and cysts. This 5 ml was mixed and transferred to a new 15-ml conical tube, and 10 ml of 3% FBS was added before being inverted 2 to 3 times to mix before centrifugation at 1,500 *× g* for 15 min at 4°C. The top 14.5 ml was then removed, and the remaining 500 μl was mixed before being transferred to a 1.5-ml Eppendorf tube. An additional 1 ml of 3% FBS was added, and tubes were inverted 2 to 3 times to mix before centrifugation at 2,000 *× g* for 15 min. Three percent FBS was carefully removed by pipette, leaving ∼200 μl of 3% FBS and cysts at the bottom of the tube. Purified cysts were then stored at 4°C until staining.

To stain and quantify cysts, a portion of whole-brain homogenate or purified cysts were pelleted before resuspension in 100 to 250 μl 3% FBS and incubated at room temperature for 1 h. Following 1 h of incubation, rhodamine-conjugated Dolichos biflorus agglutinin was added (1:500 dilution), and samples were incubated for 1 h at room temperature or 4°C overnight. Samples were then washed twice by adding 1 ml 3% FBS, inverting 6 to 10 times, and then pelleting at 2,000 × *g* for 10 min. Following the second wash, stained cysts were suspended in 200 μl, and 50-μl aliquots were placed into wells of a 96-well dish. Cysts were counted using an inverted fluorescence microscope, and the number of estimated cysts per brain was calculated based on the portion of the whole brain used for quantification.
