# Supplementary material for: Impact of Engineered Expression of Mitochondrial Association Factor 1b on Toxoplasma gondii Infection and the Host Response in a Mouse Model
Source: mSphere. 2018 Oct 17;3(5):e00471-18. doi: 10.1128/mSphere.00471-18 (PMC6193605; doi:10.1128/mSphere.00471-18)
Supplement: TABLE S1 [file sph005182663st1.pdf]

Supplemental Table 1. Summary of ANOVA applied to cytokines measured by Luminex

Cytokine	Source of variation	% of total variation	P value	Bonferroni adjusted P value*	Significant?
**G-CSF**	Interaction	28.14	< 0.0001	< 0.0032	Yes
	Row Factor (Time)	53.25	< 0.0001	< 0.0032	Yes
	Column Factor (Strain)	5.861	< 0.0001	**< 0.0032**	Yes
**Eotaxin**	Interaction	5.325	0.0218	0.6976	No
	Row Factor (Time)	67.24	< 0.0001	<0.0032	Yes
	Column Factor (Strain)	4.294	0.0024	0.0767	No
**GM-CSF**	Interaction	1.313	0.8391	> 0.9999	No
	Row Factor (Time)	32.57	< 0.0001	< 0.0032	Yes
	Column Factor (Strain)	2.555	0.1010	> 0.9999	No
**IFNγ**	Interaction	5.198	0.0004	0.0128	Yes
	Row Factor (Time)	78.42	< 0.0001	< 0.0032	Yes
	Column Factor (Strain)	1.062	0.0309	0.9888	No
**IL-1a**	Interaction	3.345	0.6498	> 0.9999	No
	Row Factor (Time)	10.07	0.1271	> 0.9999	No
	Column Factor (Strain)	0.7225	0.4669	> 0.9999	No
**IL-1b**	Interaction	4.296	0.5628	> 0.9999	No
	Row Factor (Time)	3.103	0.7064	> 0.9999	No
	Column Factor (Strain)	0.4169	0.5917	> 0.9999	No
**IL-2**	Interaction	5.828	0.3227	> 0.9999	No
	Row Factor (Time)	17.29	0.0114	0.3648	No
	Column Factor (Strain)	2.051	0.1190	> 0.9999	No
**IL-3**	Interaction	0.1847	0.9833	> 0.9999	No
	Row Factor (Time)	65.33	< 0.0001	< 0.0032	Yes
	Column Factor (Strain)	1.558	0.0763	> 0.9999	No
**IL-4**	Interaction	2.249	0.8072	> 0.9999	No
	Row Factor (Time)	7.129	0.2906	> 0.9999	No
	Column Factor (Strain)	0.4826	0.5595	> 0.9999	No
**IL-5**	Interaction	21.08	0.0002	0.0064	Yes
	Row Factor (Time)	24.74	< 0.0001	< 0.0032	Yes
	Column Factor (Strain)	1.373	0.1923	> 0.9999	No
**IL-6**	Interaction	22.74	< 0.0001	< 0.0032	Yes
	Row Factor (Time)	56.26	< 0.0001	< 0.0032	Yes
	Column Factor (Strain)	5.547	< 0.0001	**< 0.0032**	Yes
**IL-7**	Interaction	1.681	0.8354	> 0.9999	No
	Row Factor (Time)	20.82	0.0030	0.0960	No
	Column Factor (Strain)	2.009	0.1936	> 0.9999	No
**IL-9**	Interaction	6.788	0.1079	> 0.9999	No
	Row Factor (Time)	39.45	< 0.0001	< 0.0032	Yes
	Column Factor (Strain)	5.43	0.0143	0.4576	Yes
**IL-10**	Interaction	1.651	0.8222	> 0.9999	No
	Row Factor (Time)	24.61	0.0006	0.0192	Yes
	Column Factor (Strain)	3.321	0.0852	> 0.9999	No
**IL-12p40**	Interaction	2.567	0.7516	> 0.9999	No
	Row Factor (Time)	9.179	0.1589	> 0.9999	No
	Column Factor (Strain)	1.556	0.2857	> 0.9999	No
**Il-12p70**	Interaction	2.012	0.8140	> 0.9999	No
	Row Factor (Time)	15.4	0.0249	0.7968	No
	Column Factor (Strain)	0.04942	0.8452	> 0.9999	No
**IL-13**	Interaction	9.669	0.0856	> 0.9999	No

	Row Factor (Time)	17.99	0.0060	0.1920	No
	Column Factor (Strain)	4.534	0.0492	> 0.9999	No
**IL-15**	Interaction	1.432	0.8371	> 0.9999	No
	Row Factor (Time)	33.87	< 0.0001	< 0.0032	Yes
	Column Factor (Strain)	1.176	0.2818	> 0.9999	No
**IL-17**	Interaction	2.277	0.7006	> 0.9999	No
	Row Factor (Time)	30.13	< 0.0001	< 0.0032	Yes
	Column Factor (Strain)	0.6984	0.4150	> 0.9999	No
**IP-10**	Interaction	2.013	0.2719	> 0.9999	No
	Row Factor (Time)	72.45	< 0.0001	> 0.0032	Yes
	Column Factor (Strain)	3.558	0.0033	0.1056	No
**KC**	Interaction	0.8873	0.8823	> 0.9999	No
	Row Factor (Time)	50.31	< 0.001	< 0.0032	Yes
	Column Factor (Strain)	2.716e-5	0.9952	> 0.9999	No
**LIF**	Interaction	15.89	0.0025	0.08	No
	Row Factor (Time)	34.38	< 0.0001	< 0.0032	Yes
	Column Factor (Strain)	6.041	0.0102	0.3264	No
**LIX**	Interaction	5.065	0.3439	> 0.9999	No
	Row Factor (Time)	20.53	0.0024	0.0768	No
	Column Factor (Strain)	1.522	0.2452	> 0.9999	No
**MCP-1**	Interaction	8.606	0.0596	> 0.9999	No
	Row Factor (Time)	25.36	< 0.0001	< 0.0032	Yes
	Column Factor (Strain)	0.118	0.7191	> 0.9999	No
**M-CSF**	Interaction	3.482	0.5690	> 0.9999	No
	Row Factor (Time)	9.987	0.0873	> 0.9999	No
	Column Factor (Strain)	0.3091	0.6102	> 0.9999	No
**MIG**	Interaction	4.488	0.1370	> 0.9999	No
	Row Factor (Time)	56.78	< 0.0001	< 0.0032	Yes
	Column Factor (Strain)	3.492	0.0205	0.656	No
**MIP-1a**	Interaction	4.137	0.5512	> 0.9999	No
	Row Factor (Time)	8.676	0.1834	> 0.9999	No
	Column Factor (Strain)	1.213	0.3468	> 0.9999	No
**MIP-1b**	Interaction	3.051	0.2234	> 0.9999	No
	Row Factor (Time)	49.69	< 0.0001	< 0.0032	Yes
	Column Factor (Strain)	10.77	< 0.0001	**< 0.0032**	Yes
**MIP-2**	Interaction	2.789	0.5370	> 0.9999	No
	Row Factor (Time)	39.94	< 0.0001	< 0.0032	Yes
	Column Factor (Strain)	1.246	0.2397	> 0.9999	No
**RANTES**	Interaction	11.46	0.0085	0.272	No
	Row Factor (Time)	42.13	< 0.0001	< 0.0032	Yes
	Column Factor (Strain)	9.087	0.0010	**0.032**	Yes
**TNFα**	Interaction	4.115	0.0009	0.0288	Yes
	Row Factor (Time)	78.48	< 0.0001	< 0.0032	Yes
	Column Factor (Strain)	2.97	0.0002	**0.0064**	Yes
**VEGF**	Interaction	22.29	< 0.0001	< 0.0032	Yes
	Row Factor (Time)	51.17	< 0.0001	< 0.0032	Yes
	Column Factor (Strain)	10.37	< 0.0001	**< 0.0032**	Yes

*Column Factors (parasite strain) with Bonferroni correctly P values less than 0.05 are considered significant and have been **bolded**.
