# Supplementary material for: Impact of Engineered Expression of Mitochondrial Association Factor 1b on Toxoplasma gondii Infection and the Host Response in a Mouse Model
Source: mSphere. 2018 Oct 17;3(5):e00471-18. doi: 10.1128/mSphere.00471-18 (PMC6193605; doi:10.1128/mSphere.00471-18)
Supplement: TABLE S2 [file sph005182663st2.pdf]

Supplemental Table 2. Cytokines measured at or near pre-bleed levels during mouse infection with TgME49:EV or TgME49:MAF1b

		**Average Cytokine Expression (FI – background)**				
**Cytokine**	**Parasite Strain**	0 DPI	7 DPI	21 DPI	28 DPI	57 DPI
GM-CSF	TgME49:EV	-4.444	4.111	-10	-18.22	4.4
	TgME49:MAF1b	1.111	9.222	-8.333	-8.333	4.667
IL-1a	TgME49:EV	216.9	118	121.6	201.1	206.4
	TgME49:MAF1b	224	98.22	219.7	321.5	154.7
IL-1b	TgME49:EV	7.556	4.667	3.111	4.667	3.2
	TgME49:MAF1b	4.667	10.44	6.667	3.667	3.333
IL-4	TgME49:EV	73.56	21.22	7.556	2.222	10.6
	TgME49:MAF1b	26.39	20.89	4	5.333	9
IL-7	TgME49:EV	-27.94	60.89	11.67	22.78	46.2
	TgME49:MAF1b	-32.39	16.89	-9	1.5	46.33
IL-10	TgME49:EV	178.9	38.89	104.2	111.6	28.8
	TgME49:MAF1b	257.1	73.56	127	115.5	122
IL-12p40	TgME49:EV	39.22	56.44	8.444	25.11	31
	TgME49:MAF1b	62.67	52.33	33.5	47.6	22
IL-12p70	TgME49:EV	8.556	13.56	0.8889	-0.8889	10
	TgME49:MAF1b	1.611	14.89	3.333	0.3333	15.33
IL-13	TgME49:EV	111.1	58.11	105.3	95.11	77.4
	TgME49:MAF1b	175.9	46.44	92.67	93.83	232
IL-15	TgME49:EV	97.56	9.111	54.67	63.11	38.2
	TgME49:MAF1b	121.6	7.111	53.33	70.67	80.67
IL-17	TgME49:EV	12.56	24.78	8.444	3.111	12.4
	TgME49:MAF1b	7.333	32.56	10.33	5.5	20
LIF	TgME49:EV	-3.222	25.78	2.444	-1.556	20.2
	TgME49:MAF1b	-13.56	37.22	-6	8.333	132.3
LIX	TgME49:EV	5822	6298	6016	8596	14277
	TgME49:MAF1b	8126	1150	5195	6271	12551
M-CSF	TgME49:EV	5.8	14	8.6	11.4	18.8
	TgME49:MAF1b	10.4	14.1	7.714	9.143	12
MIP-1a	TgME49:EV	7.444	25.11	16.89	3.889	17.2
	TgME49:MAF1b	12.56	17.11	16.67	15	31
